# Magnesium prevents vascular calcification *in vitro* by inhibition of hydroxyapatite crystal formation

**DOI:** 10.1038/s41598-018-20241-3

**Published:** 2018-02-01

**Authors:** Anique D. ter Braake, Paul T. Tinnemans, Catherine M. Shanahan, Joost G. J. Hoenderop, Jeroen H. F. de Baaij

**Affiliations:** 1 Department of Physiology, Radboud Institute for Molecular Life Sciences, Radboud university medical center, Nijmegen, The Netherlands; 2 Institute for Molecules and Materials, Radboud University, Nijmegen, The Netherlands; 3 BHF Centre of Research Excellence, Cardiovascular Division, James Black Centre, King’s College, London, United Kingdom; 4 Department of Physiology, Anatomy and Genetics, University of Oxford, Oxford, United Kingdom

## Abstract

Magnesium has been shown to effectively prevent vascular calcification associated with chronic kidney disease. Magnesium has been hypothesized to prevent the upregulation of osteoblastic genes that potentially drives calcification. However, extracellular effects of magnesium on hydroxyapatite formation are largely neglected. This study investigated the effects of magnesium on intracellular changes associated with transdifferentiation and extracellular crystal formation. Bovine vascular smooth muscle cells were calcified using *β*-glycerophosphate. Transcriptional analysis, alkaline phosphatase activity and detection of apoptosis were used to identify transdifferentiation. Using X-ray diffraction and energy dispersive spectroscopy extracellular crystal composition was investigated. Magnesium prevented calcification in vascular smooth muscle cells. *β*-glycerophosphate increased expression of osteopontin but no other genes related to calcification. Alkaline phosphatase activity was stable and apoptosis was only detected after calcification independent of magnesium. Blocking of the magnesium channel TRPM7 using 2-APB did not abrogate the protective effects of magnesium. Magnesium prevented the formation of hydroxyapatite, which formed extensively during *β*-glycerophosphate treatment. Magnesium reduced calcium and phosphate fractions of 68% and 41% extracellular crystals, respectively, without affecting the fraction of magnesium. This study demonstrates that magnesium inhibits hydroxyapatite formation in the extracellular space, thereby preventing calcification of vascular smooth muscle cells.

## Introduction

Vascular calcifications are common in chronic kidney disease (CKD) and their presence is associated with increased cardiovascular mortality, which is the primary cause of death in CKD patients^1^. When glomerular filtration rate decreases, phosphate (Pi) levels rise and cause severely disturbed mineral and bone metabolism affecting vascular integrity and function^2^. Recently, several epidemiological studies showed a significant inverse relationship between serum magnesium (Mg^2+^) and survival in CKD patients^3–5^.

Mg^2+^ has been shown to effectively prevent mineralization in multiple experimental models of vascular calcification^6–13^. To date, experimental research has been focused on the role Mg^2+^ in the medial layer of the vessel wall, where vascular smooth muscle cells (VSMC) actively contribute to the calcification process. VSMCs transdifferentiate from contractile into osteoblast-like cells, which results in mineral deposition in the extracellular matrix, loss of contractile properties and apoptosis^14^. Typically, this process is characterized by the expression of genes normally restricted to bone tissue such as bone morphogenetic protein 2 (*BMP2*) and Runt-related transcription factor 2 (*RUNX2*) and increased alkaline phosphatase (ALP) activity^15^. Several studies suggest that Mg^2+^ directly prevents the upregulation of osteoblastic gene expression and thereby blocks VSMC transdifferentiation and subsequent mineralization^6–8,11,16,17^. However, extracellular inhibition of Mg^2+^ on calcium (Ca^2+^)-Pi particle formation, which is an important driving force for the onset of calcification, has largely been neglected^18^. Interestingly, it has been suggested that Ca^2+^-Pi nanocrystals, rather than Pi, may drive osteoblastic transdifferentation of VSMC^19^, illustrating the relevance of crystal inhibiting effects of Mg^2+^. Though poorly studied in detail in the context of VSMC calcification, the extracellular crystal inhibitory properties of Mg^2+^ are profound and well known in both biological and non-biological systems^20–22^.

This study aimed to investigate the effects of Mg^2+^ on both intracellular changes associated with vascular calcification  and crystal formation in the extracellular space. Therefore, in addition to mapping the effects of Mg^2+^ on gene expression patterns associated with osteoblastic transdifferentiation in VSMC, we studied the effects of Mg^2+^ on the formation of crystals in the extracellular space by scanning electron microscopy and X-ray diffraction.

## Materials and Methods

### Cell culture and vascular smooth muscle cell identification

Bovine aortic VSMC (bVSMC) were set up from explant culture of bovine aortic segments according to standard protocols and cultured in M199 culture medium (Lonza, Basel, Switzerland) supplemented with 10% (v/v) fetal bovine serum (FBS, HyClone, GE Healthcare Life Sciences, Illinois, USA), ciprofloxacine (Fresenius Kabi, Zeist, The Netherlands) at 37 °C in a humidified incubator with 5% (v/v) CO_2_. Standard culture medium contained 0.8 mM MgSO_4_, 1.02 mM NaH_2_PO_4_ and 1.8 mM CaCl_2_. VSMC were not used beyond the 16^th^ passage and VSMC identity was confirmed by α-smooth muscle actin (α-SMA) immunofluorescent stainings. α-SMA staining was performed on cells that were cultured on coverslips (18 mm in diameter) until 80% confluent. After fixation in 4% (w/v) paraformaldehyde and blocking with 16% (v/v) normal goat serum, the cells were stained overnight (4 °C) with a mouse monoclonal primary anti-α-SMA antibody diluted (1:400, A5228, Sigma, Missouri, USA) in a buffer containing phosphate buffered saline (PBS) and normal goat serum. Subsequently, cells were stained with a goat-anti mouse IgG Alexa 488 conjugated polyclonal antibody (1:250, A-11029, Invitrogen, Massachusetts, USA) and visualized using an Axio Imager M (Zeiss, Oberkochen, Germany).

### Experimental design

For calcification, medium was supplemented with 5% (v/v) FBS and 10 mM *β*-glycerophosphate (BGP, Merck Millipore, Massachusetts, USA). BGP requires cellular activity for its cleavage to free Pi, and was chosen in this setup to increase the medium Pi concentration to minimize Mg^2+^-Pi interactions prior to cellular exposure. In the high Mg^2+^ treatment medium, MgCl_2_ (Merck Millipore) was supplemented to reach a final concentration of 2 mM MgCl_2_. At 80% confluence, cells grown in a 12-wells plate were incubated with designated media for 14 days, which was changed every 2–3 days.

### TRPM7 inhibition by 2-APB

Transient receptor potential melastatin 7 (TRPM7) was inhibited by incubation with 10 μM of 2-Aminoethyl diphenylborinate (2-APB, Sigma). At 80% confluence, 2-APB was supplemented in combination with the different culture media as outlined in the previous section during 14 days in a 24-wells plate.

### Pi concentration of the cell culture supernatant

Pi was measured in the cell culture supernatant (or culture medium) calorimetrically using the malachite green method as described elsewhere^23^. Briefly, a reaction mix consisting of molybdate and malachite green was added to the samples and standards and incubated for 30 minutes at room temperature. The absorbance was measured at 620 nm using a Benchmark Plus Microplate Spectrophotometer System (Bio-Rad, California, USA).

### Quantification of Ca^2+^ deposition

Cells were decalcified with 0.1 M HCl for 5 minutes at room temperature with gentle rocking, which effectively dissolved all Ca^2+^ deposits present. The Ca^2+^ concentration in the supernatant was determined by the *o*-cresophthalein complexone method. *o*-cresophthalein color reagent (Sigma) was incubated with the samples and standards and the absorbance was measured immediately at 570 nm, as described previously^24^. Subsequently, the cells were neutralized in PBS and lysed in 0.1 M NaOH/0.1% (w/v) sodium dodecyl sulfate for total protein isolation. Ca^2+^ concentrations were normalized for total protein as determined by Pierce BCA protein detection kit according to the manufacturer’s instructions (Fisher Scientific, Massachussets, USA).

### Alizarin Red staining

Calcification was visualized by the Alizarin Red S staining method for Ca^2+^. Cultures were washed with PBS, fixed in 4% (v/v) formalin for 15 minutes and washed with demineralized H_2_O before staining with 2% (w/v) aqueous Alizarin Red (Sigma) for 5 minutes.

### RNA isolation and real-time polymerase chain reaction (RT-qPCR)

Total RNA was extracted from VSMC using TRIzol (Invitrogen) and treated with DNAse (1 U/μg RNA, Promega, Wisconsin, USA) to remove genomic DNA. cDNA was synthesized from 1.5 μg total RNA by Moloney Murine Leukemia Virus reverse transcriptase (Invitrogen) for one hour at 37 °C. The primers used for PCR amplification are shown in Table 1 and were equally efficient. RT-qPCR was executed in duplicate using IQ SYBRGreen Mix according to the manufacturers protocol (Bio-Rad), using a Bio-Rad thermos-cycler (Bio-Rad). The expression of target genes was normalized to *GAPDH* expression levels.

**Table 1:** Bovine qPCR primer sequences.

**Gene (** ***Bos Tauros)***	**Forward primer sequence**	**Reverse primer sequence**
*GAPDH*	5′-AAGATTGTCAGCAATGCCTCC-3′	5′-TGGACAGTGGTCATAAGTCCC-3′
*ACTA2*	5′-TCTTTGAAGGCAAAGACCTGG-3′	5′-ATTCCCTCTTATGCTCCTGGG-3′
*RUNX2*	5′-AGGCGCATTTCAGATGATGAC-3′	5′-ACCTGCCTGGCTCTTCTTAC-3′
*BMP2*	5′-GCAGCTTCCATCACGAAGAATC-3′	5′-CCGAAAGACCTGAAGTTCTGC-3′
*ALPL*	5′-ACCTCCGTAGAAGACACACTG-3′	5′-GCCAGACCAAAGATCGAGTTG-3′
*TRPM7*	5′-GTCGTATGTGAAGGAACAGGC-3′	5′-TGCATCAGGAAGATTCCCTCC-3′
*MGP*	5′-TGGCAGCTCTGTGTTATGAATC-3′	5′-GGCTTTTGCTCTCCATCTCTG-3′
*OPN*	5′-CTAACGTTCAGAGTCCAGATGC-3′	5′-TTGGAAAGCTCGCTACTGTTG-3′
*OPG*	5′-AAAGCGCCCTGTAGAAAACAC-3′	5′-ACAGGGTCATGTCTATTCCGC-3′

### Alkaline phosphatase activity assay

Cells grown in 12-well plates and cultured in designated media for 2, 8 and 14 days were lysed with 1% (v/v) Triton X-100 in PBS containing protease inhibitors. ALP activity was determined in the total lysate as the hydrolysis of *p*-nitrophenyl phosphate (sigma) into *p*-nitrophenol in a basic buffer by ALP by *p*-nitrophenol production. The reaction was incubated for 30 minutes at 37 °C and the absorbance for *p*-nitrophenol was measured at calorimetrically at 410 nm for both *p*-nitrophenol (Sigma) standards and samples. One unit (U) was defined as the production of 1 µmol *p-*nitrophenol per minute per gram protein.

### Detection of apoptosis

Occurrence of apoptosis was measured using the Annexin V-FITC Apoptosis Detection Kit (ab14085 Abcam, Cambridge, UK). Due to the presence of calcifications, the method for adherent cells was used according to the manufacturer’s instructions. Briefly, cells were incubated with Annexin-V-FITC and propidium iodide in binding buffer for 5 minutes at room temperature. Apoptotic cells were detected by immunofluorescence microscopy using a FITC and Texas Red filter on an Axio Imager M (Zeiss). Apoptosis was quantified using ImageJ software (NIH, Maryland, USA) by calculating the ratio of the area positive for FITC signal versus total area, as the mean of multiple captures in 3 replicates per treatment per time-point.

### Crystal isolation for X-ray diffraction, scanning electron microscopy and energy-dispersive spectroscopy analysis

Cell culture supernatants of BGP-treated cells in 6-well plates with and without 2 mM MgCl_2_ were collected and purified. Supernatants were centrifuged for one hour at 16 000 × *g*^19^. The pellets containing the nanocrystals were washed with demineralized H_2_O and then re-centrifuged. Subsequently, the crystal pellets were dried and used for analysis. One measurement represents the crystals formed in a total of 7 wells containing 2 ml of culture medium in the BGP treated cultures. As less material was formed in the BGP cultures supplemented with Mg^2+^, one measurement represents the crystals formed in a total of 21 wells containing 2 ml of culture medium in order to reach sufficient amounts to detect by X-ray diffraction. For X-ray diffraction analysis, diffractograms were measured on a PANalytical Empyrean (PANalytical, Almelo, the Netherlands) in transmission mode with fine-focus sealed tube, focusing mirror and PIXcel3D detector, using CuKα radiation. The samples were measured in a capillary, using 0.5 mm soda glass capillaries with a wall thickness of 0.01 mm. For scanning electron microscopy (SEM) (GeminiSEM, Zeiss) in combination with energy-dispersive spectroscopy (EDX) for elemental analysis (QUANTAX 200, Bruker) the crystal pellets were transferred onto copper tape and coated with carbon. High-resolution pictures were obtained using an Everhart-Thornley SE detector. Accelerating voltage was 5 kV for morphological observations and 15 kV for micro-elemental analyses. Due to the use of BGP as calcification inducer, a cell-free control could not be included as cellular presence is necessary to cause Pi accumulation in the medium (data not shown).

### Statistics

Parametric data were analyzed by One-Way ANOVA with Tukey’s post-hoc test to correct for multiple comparisons using PRISM software (GraphPad, San Diego, CA). Non-parametric data as identified by Shapiro-Wilk test for normality were analyzed using Kruskall-Wallis analysis with Dunn’s correction for multiple analysis. Time-course data was analyzed using a Two-Way ANOVA. All data are shown as mean ± SEM. *P* < 0.05 was considered statistically significant.

## Results

### *β*-glycerophosphate supplementation resulted in increased medium Pi concentration

**Figure 1:**
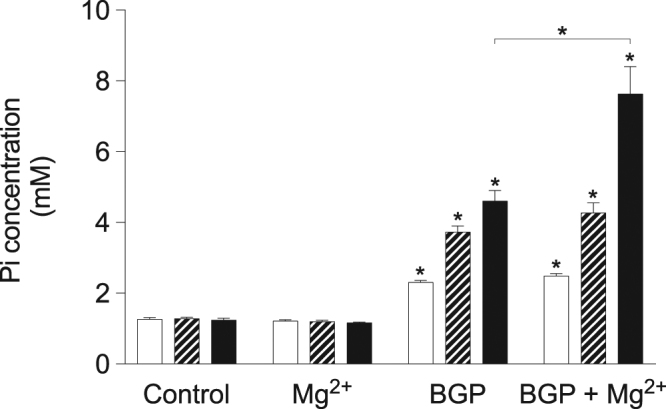
BGP increased medium Pi concentration. bVSMC were cultured with 10 mM BGP and the Pi concentration in the cell culture supernatant was measured in whole media at 2 (white bars), 8 (striped bars) and 14 (black bars) days. Data are shown as the mean of 3 individual experiments (each consisting of 3 replicates) ±SEM. Unless shown otherwise, *Indicates *P* < 0.05 versus control. BGP, *β*-glycerophosphate; bVSMC, bovine vascular smooth muscle cells; Pi, inorganic phosphate; SEM, standard error of the mean.

BGP is a Pi-donor that requires enzymatic cleavage in order to release Pi. As Pi exposure is one of the decisive factors in the calcification process, the Pi concentration of the cell culture supernatant was assessed after 2, 8 and 14 days of treatment (Fig. 1). 10 mM BGP treatment resulted in gradual increase in Pi concentration over time, reaching 4.6 ± 0.3 mM after 14 days. 2 mM Mg^2+^ supplementation led to significantly higher Pi concentrations of 7.6 ± 0.8 mM after 14 days. In contrast, BGP treatment in cell-free conditions did not lead to increased Pi concentrations under the same conditions (data not shown).

### Mg^2+^ prevents vascular smooth muscle cell  mineralization

**Figure 2:**
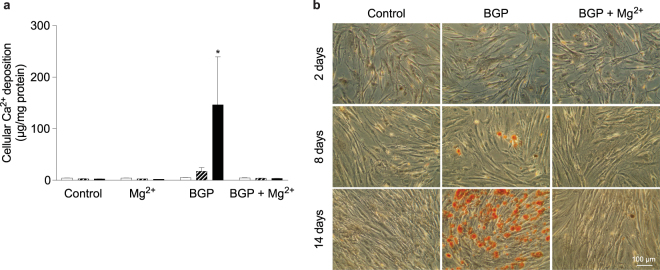
High Mg^2+^ prevented mineralization, despite high medium Pi concentrations. Quantification of calcification by cellular calcium deposition at 2 (white bars), 8 (striped bars) and 14 (black bars) days during calcification by BGP supplementation (**a**) and Alizarin Red staining for calcium (**b**). Calcium deposition data are shown as the mean of 3 individual experiments (each consisting of 3 replicates) ±SEM. *Indicates *P* < 0.05 versus control. Scale bar corresponds with 100 µm. BGP, *β*-glycerophosphate; Pi, inorganic phosphate; SEM, standard error of the mean.

The effect of BGP on the development of calcifications was studied using cellular Ca^2+^ measurements and visualized by Alizarin Red staining (Fig. 2). 10 mM BGP treatment of bVSMC resulted in variable but pronounced calcification after 14 days (146 ± 93 versus 2.5 ± 0.2 µg Ca^2+^ per gram protein in the control condition, p < 0.05). Ca^2+^ deposition was completely prevented by 2 mM Mg^2+^ in our model (3.5 ± 0.3 µg/g protein Ca^2+^).

### *β*-glycerophosphate supplementation upregulated OPN gene expression but did not result in changes in mRNA expression of calcification activators

**Figure 3:**
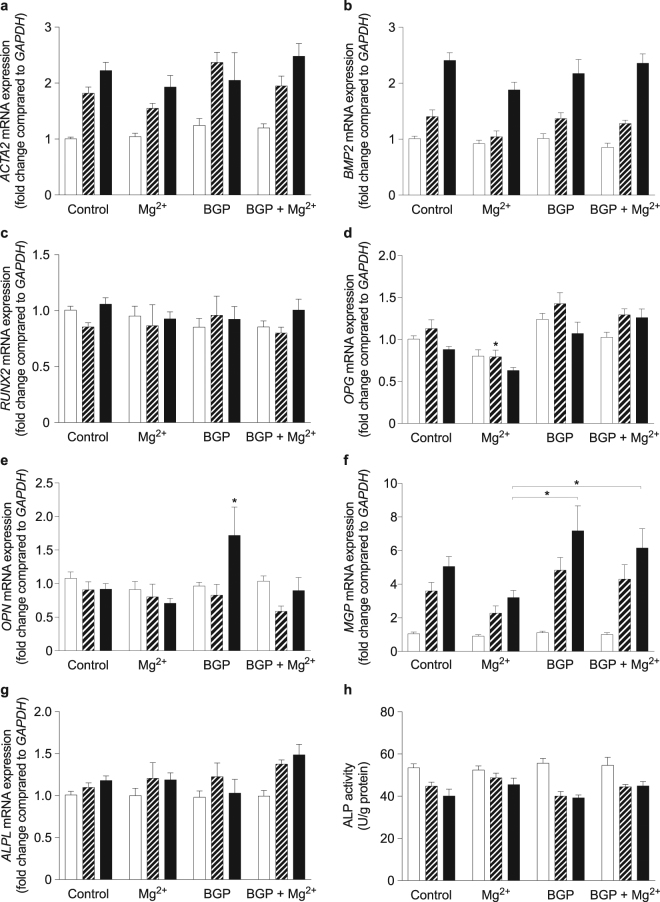
Bovine VSMC did not underdo osteogenic transdifferentiation after BGP treatment. mRNA expression of *ACTA2* (**a**), osteogenic transcription factors *RUNX2* (**b**) and *BMP2* (**c**) and calcification inhibitors OPG (**d**), OPN (**e**) and MGP (**f**) were measured after 2 (white bars), 8 (striped bars) and 14 (black bars) days of BGP supplementation in presence or absence of Mg^2+^. mRNA levels were normalized for *GAPDH* expression and are shown relative to the 2-day control. ALP activity (**g**) and mRNA expression (**h**) remained stable during BGP treatment. Data are shown as the mean of 3 individual experiments (each consisting of 3 replicates) ±SEM. Unless shown otherwise, significance is indicated versus control (*Indicates *P* < 0.05). ACTA2, α-smooth muscle actin; ALPL, alkaline phosphatase; BGP, *β*-glycerophosphate; BMP2, bone morphogenetic protein 2; bVSMC, bovine vascular smooth muscle cells; GAPDH, glyceraldehyde 3-phosphate dehydrogenase; MGP, matrix gla protein; OPG, osteoprotegerin; OPN, osteopontin; RUNX2, Runt-related transcription factor 2; SEM, standard error of the mean.

To assess the effects of Mg^2+^ involved in the prevention of VSMC calcification, gene expression levels of *ACTA2* and osteogenic transcription factors *RUNX2* and *BMP2* were assessed after 2, 8 and 14 days (Fig. 3a–c). 10 mM BGP treatment did not result in expression changes of *ACTA2*, *RUNX2* and *BMP2* over the time course of calcification. 2 mM Mg^2+^ supplementation did not affect these expression levels. Both *ACTA2* and *BMP2* mRNA expression significantly increased over time in all conditions, independent of BGP treatment (Fig. 3a–c). In addition, mRNA expression levels of calcification inhibitors *OPG*, *OPN* and *MGP* were assessed (Fig. 3d–f). *OPG* expression was significantly downregulated by Mg^2+^ supplementation compared all other treatments after 8 days. No effect of BGP was observed at all time-points. *MGP* gene expression showed a significant increase over time in all treatment groups, while no effect of BGP was observed. After 14 days, BGP significantly increased *OPN* expression, which was prevented by Mg^2+^. In addition to osteogenic transcription factors, ALP is a well-known indicator for osteoblastic maturation. Therefore, ALP mRNA expression and activity were measured to assess the effect of Mg^2+^ on the development of an osteoblastic phenotype after 2, 8 and 14 days. BGP did not change *ALPL* mRNA expression and ALP activity, which remained stable after Mg^2+^ supplementation (Fig. 3g,h).

### VSMC mineralization preceded apoptosis

**Figure 4:**
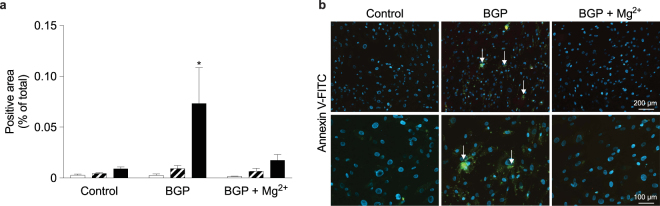
Calcification preceded bVSMC apoptosis. Apoptosis was detected on adherent cells by Annexin-V FITC conjugated antibody quantified at 2 (white bars), 8 (striped bars) and 14 (black bars) days of BGP supplementation in presence or absence of Mg^2+^ (**a**). Nuclei are stained with DAPI (blue), Annexin-V positive cells (green) indicate apoptosis (arrows), which was observed only after onset of calcification at 14 days. Images represent 3 individual experiments. Scale bars correspond to 200 µm (upper panel) and 100 µm (lower panel). Prior to calcification, as represented by 2-day and 8-day treated samples, no apoptosis was detected (**b**). *Indicates *P* < 0.05 versus control. BGP, *β*-glycerophosphate.

As apoptosis accelerates VSMC calcification *in vitro*^25^, the occurrence of apoptosis was assessed in VSMC at 2, 8 and 14 days (Fig. 4). 10 mM BGP treatment did not result in increased apoptosis before the onset of calcification at 2 and 8 days. After 14 days, apoptosis was observed in calcified regions. Mg^2+^ prevented calcification at all time-points as apoptosis was not detected in the Mg^2+^-supplemented BGP cells.

### TRPM7 was not involved in the protective effect of Mg^2+^ against *β*-glycerophosphate-induced calcification

**Figure 5:**
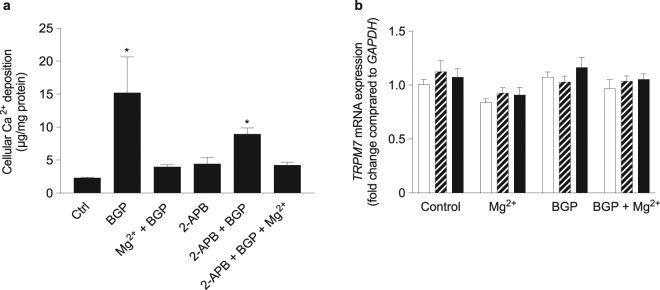
Inhibition of TRPM7 did not abrogate the protective effects of Mg^2+^. Ca^2+^ deposition was measured in calcifying bVSMC supplemented with Mg^2+^ cultured with 10 µM 2-APB, a TRPM7 inhibitor, for 14 days (**a**). In calcifying bVSMC, mRNA expression levels of *TRPM7* were not affected after 2 (white bars), 8 (striped bars) and 14 days (black bars) (**b**). Data are shown as the mean of 3 individual experiments (each consisting of 3–4 replicates) ± SEM. *Indicates *P* < 0.05 versus control. 2-APB, 2-Aminoethyl diphenylborinate; BGP, *β*-glycerophosphate; bVSMC, bovine vascular smooth muscle cells; TRPM7, transient receptor potential melastatin.

TRPM7 is the main Mg^2+^ channel in VSMCs^26^. To examine whether a TRPM7-mediated increasing intracellular Mg^2+^ concentration prevents VSMC calcification, VSMCs were treated with 10 µM 2-APB, which is a TRPM7 blocker^11^. 2-APB treatment of Mg^2+^ supplemented BGP cultures did not reverse the protective effect of Mg^2+^ on BGP-induced VSMC calcification (Fig. 5a). Furthermore, 10 mM BGP treatment did not affect TRPM7 mRNA expression levels in cultured VSMC (Fig. 5b).

### Mg^2+^ prevented *β*-glycerophosphate-induced formation of extracellular hydroxyapatite crystals

**Figure 6:**
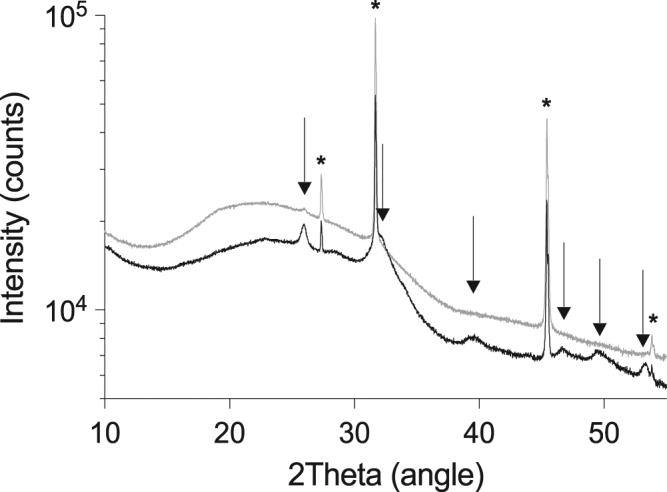
Impact of Mg^2+^ on BGP-induced Ca^2+^-apatite formation. X-ray powder diffraction analysis of cell culture supernatants show presence of NaCl (*) and Ca-apatite (arrows) in BGP treated cultures (black line), but not in BGP cultures supplemented with Mg^2+^ (gray line). Of note: as the BGP culture supplemented with Mg^2+^ contained less precipitates; one measurement represents 21 cultures (6-well format) compared to 7 in the BGP condition in order to reach the detection threshold. BGP, *β*-glycerophosphate.

**Figure 7:**
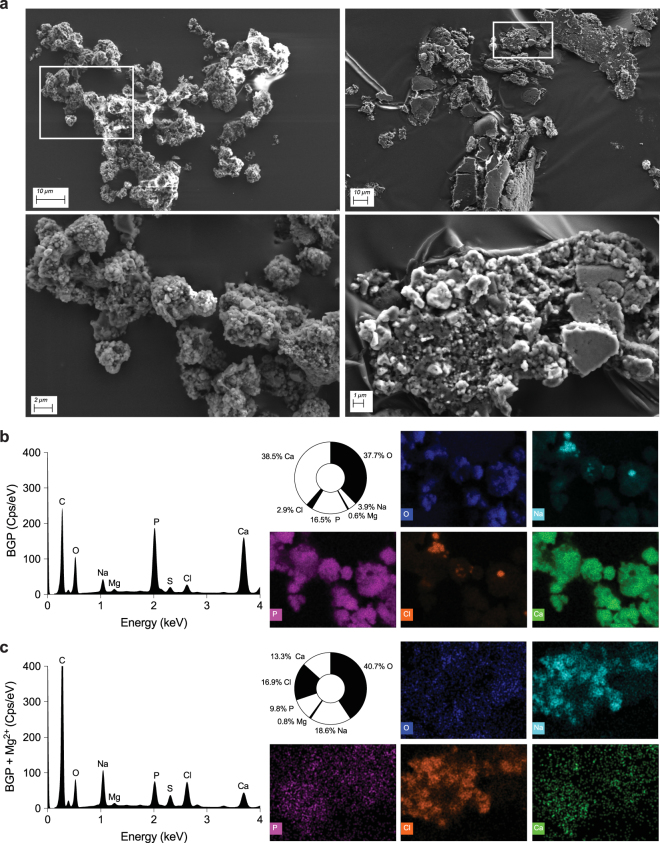
Crystal or particle clusters formed in BGP cell culture supernatant are morphologically and chemically distinct. Morphology of crystal clusters formed in BGP and in BGP supplemented with Mg^2+^ cell culture supernatants, as overview (upper) and focused (lower) for EDX analysis (**a**). The averaged EDX-spectrum and resulting quantification of the crystal cluster isolated from Mg^2+^ supplemented culture supernatant reveals reduced Ca (green) and P (purple), as visualized in the elemental map (**c**) compared to crystal clusters found in BGP treated cultures (**b**). In addition, Mg^2+^ supplemented crystal clusters showed increased Na (blue) and Cl (orange) compared to BGP crystal clusters. Data are presented as mean of the analyses of ten individual crystal clusters. BGP, *β*-glycerophosphate; CPS, counts per second; EDX, energy dispersive spectroscopy; (k)eV, (kilo)-electron volt.

To investigate the potential role of Mg^2+^ in crystal growth and formation, the cell culture supernatants of BGP-treated cells in the presence or absence of Mg^2+^ were analyzed for the incidence of crystals using X-ray powder diffraction. In the BGP-treated samples, the X-ray diffraction patterns revealed the presence of a considerable amount of hydroxyapatite crystals (Fig. 6). The broadening of the hydroxyapatite diffraction peaks, compared to the NaCl peaks, indicate that the hydroxyapatite crystals are nano-sized. Both crystals isolated from the cell culture supernatants and a synthetic hydroxyapatite standard, that was used as positive control, matched with a reference diffraction pattern specific for hydroxyapatite crystals (see Supplemental Fig. 2). Hydroxyapatite diffraction peaks were absent in the Mg^2+^-supplemented BGP supernatants. Of note, no crystals other than hydroxyapatite and NaCl were identified. As X-ray diffraction exclusively detects crystalline material and not amorphous material, isolated particles were analyzed by SEM-EDX for morphology and elemental composition (Fig. 7). EDX analysis revealed a reduced Ca^2+^ and Pi fraction in crystal clusters of 68% and 41% after Mg^2+^ supplementation, respectively, without increasing the fraction of Mg^2+^ present in the crystal clusters (Fig. 7b,c).

## Discussion

Here, we demonstrate that Mg^2+^ inhibits bVSMC mineralization through inhibition of Ca-apatite formation in the extracellular space, independent of VSMC transdifferentiation. Our most important finding is the absence of hydroxyapatite crystals in the medium of BGP-treated bVSMCs supplemented with Mg^2+^. Characterization by SEM-EDX confirmed the reduction of Ca-apatite crystals in Mg^2+^-supplemented supernatants, without incorporation of Mg^2+^ in the formed crystals. Underlining the strong capacity of Mg^2+^ to block crystal formation, 2 mM Mg^2+^ was sufficient to prevent the calcification process, even though extracellular Pi levels rise to 7 mM in Mg^2+^-BGP treated bVSMCs. Moreover, intracellular action of Mg^2+^ is not likely in our setup, because when cellular Mg^2+^ uptake was impaired using TRPM7-blocker 2-APB, Mg^2+^ still prevented vascular calcification^11^. Accordingly, we propose that Mg^2+^ prevents VSMC mineralization through the inhibition of hydroxyapatite formation in the extracellular space, blocking its deposition on VSMC.

Hydroxyapatite (Ca_10_(PO_4_)_6_(OH)_2_) is the most abundant type of crystal in uremic arterial calcifications and its formation has been shown to be essential for VSMC transdifferentiation and vascular calcification^19,27^. Although it has been suggested that the potential incorporation of Mg^2+^ in Ca-apatite crystals (whitlockite, Ca_9_Mg(HPO_4_)(PO_4_)_6_), may reduce crystal pathogenicity and increase solubility, we did not identify any whitlockite after Mg^2+^ treatment. Our data suggest that Mg^2+^ most likely prevents crystal nucleation, rather than affecting crystal content. These findings are in line with previous studies that exclusively identified hydroxyapatite, and not whitlockite, in deposits on calcifying VSMC supplemented with Mg^2+^ ^28^. Indeed, high concentrations of Mg^2+^ led to less hydroxyapatite deposition in a study by Louvet *et al.*^29^. Given the lack of crystals after Mg^2+^ supplementation, our results indicate that Mg^2+^ inhibits the early phases of crystal assembly in high Pi-media. Moreover, we hypothesize that the incorporation of Pi in hydroxyapatite in the BGP condition explains the lower free Pi concentration in BGP-treated compared to the Mg^2+^-BGP-treated culture media.

Initial Ca-Pi particle formation in response to elevated Pi-levels has shown to occur in a cell-independent manner, subsequently initiating VSMC transdifferentiation when native VSMC inhibitory capacities diminish^19,30^. Though poorly studied in the context of VSMC mineralization, Mg^2+^ is known to stabilize amorphous Ca-Pi particles and therefore inhibit Ca-apatite maturation in acellular systems^31–36^. While the exact mechanisms remain unknown, evidence suggests that Mg^2+^ may stabilize extracellular ATP. Hydrolysis of ATP is necessary for hydroxyapatite nucleation^21^. The importance of crystal maturation in the initiation of VSMC transdifferentiation and vascular calcification has been frequently emphasized^18,19,30,37^. Recently, Ca-Pi-containing soluble nanoparticles or calciprotein particles (CPP) were shown to stimulate calcification^37^. Interestingly, Mg^2+^ delays CPP maturation in uremic serum^38^. These findings support that Mg^2+^ prevents mineralization by directly inhibiting Ca-apatite crystal formation or maturation in the extracellular space.

Mg^2+^ supplementation has repeatedly shown to prevent osteogenic gene expression. As a result, osteogenic gene expression has been repeatedly considered as Mg^2+^ target to prevent osteoblastic transdifferentiation^39^. In line with this hypothesis, previous research showed that Mg^2+^ concentrations as low as 0.8 mM reversed established calcification in human VSMC, which could be abrogated by 2-APB treatment^17^. These results suggest that cellular Mg^2+^ uptake via TRPM7 prevents VSMC calcification^7,11,17^. However the role of TRPM7 is controversial, as recent evidence suggests that interleukin-18 enhanced VSMC calcification through TRPM7 activation^40^. In our model, TRPM7 inhibition by 2-APB did not affect the Mg^2+^ rescue.

bVSMC are characterized by high basal expression levels of ALP, which makes them prone to calcification. Despite their susceptibility to calcify, the bVSMCs are contractile and do not present any signs of osteoblastic transdifferentiation, as high levels of *ACTA2* expression and α-SMA protein expression were preserved in response to BGP supplementation. Although our bVSMCs strongly calcified, BGP treatment did not result in osteogenic conversion as demonstrated by stable expression of *BMP2*, *RUNX2* and *ALPL* among treatments^14^. Interestingly, both mRNA expression of *BMP2* and *ACTA2* increased over time. However, these observations were irrespective of treatment and are therefore not related to osteoblastic transdifferentiation of the bVSMC. The only transcriptional response observed during BGP-induced calcification was upregulation of the *OPN* gene after 14 days, which was prevented by Mg^2+^. Increased *OPN* expression is associated with calcification^19,41,42^. *OPN* is an inhibitor of calcification and potently inhibits hydroxyapatite growth and *OPN* upregulation has been shown to reflect a protective mechanism in response to the phosphate- and hydroxyapatite-rich environment by VSMC^43–45^. The absence of *OPN* upregulation in Mg^2+^-supplemented BGP cultures may therefore be explained by the lack of Ca-Pi formation. Moreover, *OPN* is only increased at 14 days after calcification was already manifested, suggesting it to be resulting from calcification rather than causing. In addition to osteoinductive signaling, apoptosis has been shown to induce the progression of calcification^25^. Our results indicate that calcification precedes apoptosis, as apoptosis was only detected after 14 days of BGP treatment when calcification was already manifested. In our setup, apoptosis is likely the result of exposure to Ca-Pi crystals, rather than a causative factor for calcification^46^.

In human, rodent and bovine calcification models evidence strongly suggests that calcification is a result of VSMC undergoing osteogenic transdifferentiation and that Mg^2+^ effectively abrogates this through upregulation of calcification inhibitors and downregulation of osteogenic genes^6–8,11,16,17,47,48^. Indeed, we show the effective inhibition of Mg^2+^ in VSMC calcification. However, in contrast to previous studies, our results suggest that calcification is driven by extracellular hydroxyapatite formation independent of osteogenic transdifferentiation in bVSMCs. While many studies show the association between osteogenic transdifferentiation and vascular calcification, it remains debatable whether this transdifferentiation is an undisputable prerequisite for the development of mineralization^49^. Calcification represents the final common pathway of multiple pathological vascular processes^50^. Our results do not contradict intracellular Mg^2+^ effects on osteoblastic transdifferentation. However, they do highlight the presence of alternative extracellular effects on crystal formation. Overall, it is important to note that potential intracellular and extracellular pathways involved in the calcification-inhibiting capacity of Mg^2+^ are not mutually exclusive. Given the strong effect of Mg^2+^ on calcification independent of osteogenic pathways, however, the relative contribution of crystal inhibition compared to any intracellular targets may be considerable and underestimated to date.

An important strength of this study is that our model favored to study the effects of Mg^2+^ on extracellular crystal growth, independent of genetic VSMC transdifferentiation. Although it has been reported previously that Mg^2+^ inhibits calcification, most study set-ups do not allow to discriminate between extracellular reduction of crystal formation and intracellular inhibition of osteogenic conversion^6–8,11,17^. A limitation of this study is that while we show that TRMP7 seems not to be involved in calcification, we cannot exclude that other Mg^2+^ channels than TRPM7 facilitate Mg^2+^ entry into the bVSMCs. However, it was shown previously that TRPM7 is the main Mg^2+^ channel in VSMCs^26^. Therefore, the contribution of other transporters is likely minor. In addition, we show the effectiveness of Mg^2+^ primarily through extracellular mechanisms involving hydroxyapatite. In contrast to other studies, the driving force of calcification in our model is not osteoblastic transdifferentiation but mainly hydroxyapatite formation and deposition. Therefore, any intracellular effects of Mg^2+^ involving modification of osteogenic genes such as *BMP2* and *RUNX2* cannot be excluded.

In conclusion, our findings demonstrate a role for Mg^2+^ in preventing VSMC mineralization involving direct extracellular Ca-apatite crystal inhibition. An increasing body of studies now report that Mg^2+^ prevents vascular calcification by extracellular Pi binding. Mg^2+^ has been shown to reduce both vascular and non-vascular calcifications and to improve calcification propensity, favoring a non-cellular mechanism of action^38,51^. Therefore, Mg^2+^ may be considered an important and realistic approach to potentially reduce the risk for vascular calcification and subsequent cardiovascular complications in CKD patients. Clinical trials are warranted to further assess the clinical relevance of Mg^2+^ in relation to vascular calcifications.

## Electronic supplementary material

Supplementary Figures
