# Supplementary material for: Magnesium prevents vascular calcification in vitro by inhibition of hydroxyapatite crystal formation
Source: Sci Rep. 2018 Feb 1;8:2069. doi: 10.1038/s41598-018-20241-3 (PMC5794996; doi:10.1038/s41598-018-20241-3)
Supplement: Supplementary file 1 — Supplementary Figures [file 41598_2018_20241_MOESM1_ESM.pdf]

TITLE PAGE

Magnesium prevents vascular calcification *in vitro* by inhibition of hydroxyapatite crystal formation

Anique D. ter Braake[1], Paul T. Tinnemans[2], Catherine M. Shanahan[3], Joost G. J. Hoenderop[1], Jeroen H.F. de Baaij[1,4]

[1]Department of Physiology, Radboud Institute for Molecular Life Sciences, Radboud university medical center, Nijmegen, The Netherlands

[2]Institute for Molecules and Materials, Radboud University, Nijmegen, The Netherlands

[3]BHF Centre of Research Excellence, Cardiovascular Division, James Black Centre, King's College, London, United Kingdom

[4]Department of Physiology, Anatomy and Genetics, University of Oxford, Oxford, United Kingdom

Corresponding author:

Dr. Jeroen H.F. de Baaij

Department of Physiology (286), Radboud Institute for Molecular Life Sciences

Radboud university medical center

P.O. Box 9101, 6500 HB Nijmegen, The Netherlands

Email: jeroen.debaaij@radboudumc.nl

Phone: +31- 24 36 17510

Keywords: Chronic kidney disease, hydroxyapatite, magnesium, vascular calcification, vascular smooth muscle cells

Supplementary figures and legends

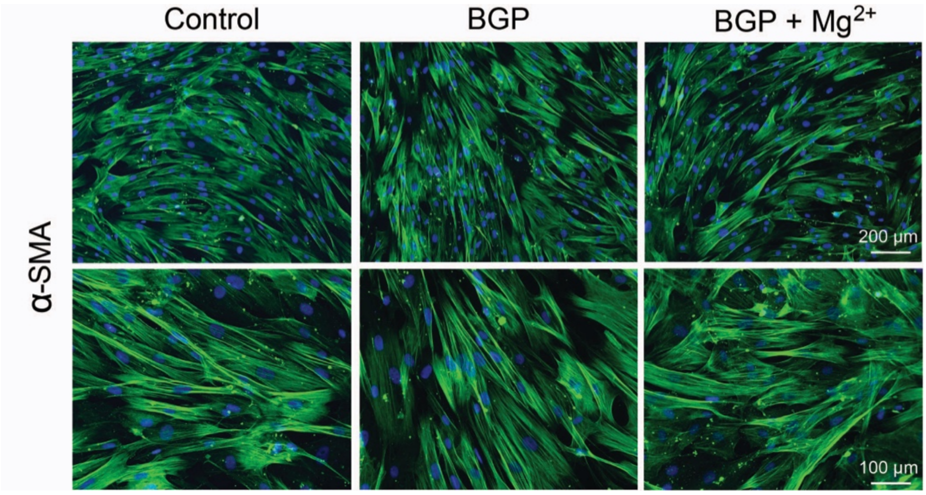

Supplemental figure 1 BGP treated bVSMC show preserved α-SMA protein expression. Smooth-muscle alpha actin (α-SMA) protein expression in cultures treated with BGP for 14 days was assessed by immunofluorescence (green). Nuclei are stained with DAPI (blue). Scale bars correspond with 200 µm (upper panel) and 100 µm (lower panel).

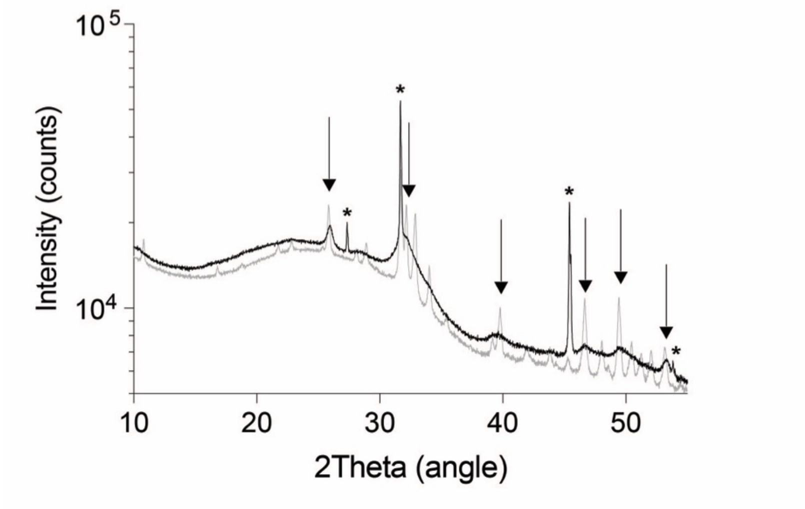

Supplemental figure 2 X-ray diffraction patterns of BGP-induced crystals and commercial hydroxyapatite standard. Comparative X-ray powder diffraction analysis of crystals in BGP treated cultures (black line) and commercially available hydroxyapatite crystal (gray line). Ca-apatite diffraction peaks are indicated by the arrows and NaCl by *.
